# Effects of exercise training on type 2-diabetes: the role of Meteorin-like protein

**DOI:** 10.15171/hpp.2019.12

**Published:** 2019-05-25

**Authors:** Ayoub Saeidi, Seyed Morteza Tayebi, Abedin Khosravi, Fatemeh Malekian, Arash Khodamoradi, Maha Sellami, Abderraouf Ben Abderrahman, Hassane Zouhal

**Affiliations:** ^1^Exercise Biochemistry Division, Faculty of Physical Education and Sport Science, University of Mazandaran, Babolsar, Iran; ^2^Core Research of Health Physiology and Physical Activity, Department of Exercise Physiology, Faculty of Sport Science, Allameh Tabataba’i University, Tehran, Iran; ^3^Department of Physical Education and Sport Science, Payame Noor University, Tehran, Iran; ^4^Southern University, Agricultural Land Grant Campus, Baton Rouge, Louisiana, USA; ^5^University of Qatar, Doha, Qatar; ^6^Faculty of Sciences of Bizerte, University of Carthage, Zarzouna, Tunisia; ^7^Movement, Sport and Health Sciences laboratory (M2S), University of Rennes 2, Rennes, France

## Dear editor

Diabetes, as a chronic condition affects millions of people worldwide. In the United States, approximately 84 million people have pre-diabetes, a condition that blood sugar is elevated but not to the number that is considered as diabetic.^1-3^ More than 30 million people in the United States are diabetic which causes an enormous pressure on individuals, public health, and economics.^1^ International Diabetes Federation (IDF) has predicted that the number of cases of type 2 diabetes in the Middle East will increase to 40 million (80% increase) in 2025.^4^ In Iran, more than 3 million people are suffering from type 2-diabetes, which has tripled every 15 years.^5^ Type 2-diabetes is caused by a combination of genetic disorders and environmental factors such as lack of physical activity and poor nutrition.^6^ Obesity plays a key role in most cases of type 2-diabetes. When people become more obese, they reach an abnormal insulin resistant state, causing impaired glucose tolerance, which ultimately leads to the onset of type 2-diabetes. People with type 2-diabetes are at risk of complications including retinopathy and neuropathy. In some cases, type 2-diabetes could be the cause for blindness, kidney failure, and even heart attacks.^1^ In diabetic people tissues such as liver, skeletal muscle, and adipose tissue are not able to pick up insulin from blood circulation so over time this leads to insulin resistance. However, this can be compensated by a large amount of insulin production through pancreas.^7^ In the past, type 2-diabetes usually has targeted adults but the number of children and adolescents affected by this disease has been increased dramatically. The main reasons could be poor nutrition and sedentary lifestyle.

Exercise and physical activities can benefit the organs and can protect the body against obesity and type 2-diabetes.^8,9^ Secreted proteins can play a critical role in both physiological and pathological processes and could be utilized as biomarkers and treatments for chronic conditions such as aging. Meteorin-like protein (Metrnl) is a novel secreted protein homologous to the neurotrophin Metrn. However, this protein, unlike Metrn present in the brain, shows a higher distribution expression in white adipose tissue and barrier tissues in the brain compared to others. Metrnl plays important roles in neural regeneration, turning white adipose to brown and insulin sensitization. Metrnl is also called Cometin, Subfatin and Interleukin 39, referred to its neurotrophic effects, adipokine functions and probably as a cytokine, respectively. Up to date, the spectrum of Metrnl functions and mechanism need to be investigated.^10^ Previous study has showed that adipocyte Metrnl controls insulin sensitivity by the peroxisome proliferator-activated receptor gamma (PPAR) pathway, acts as an insulin sensitizer and possibly a therapeutic target for insulin resistance.^11-13^ Another report argued that up-regulation in peroxisome proliferator-activated receptor gamma coactivator-1 (PGC-1), as an important regulator that induces mitochondrial biogenesis and increases Metrnl in muscle tissue. Metrnl could also transmit the positive effects of PGC-1 to other tissues followed by an up-regulation.^14^

A recent study represented Metrnl as an adipokine abundantly present in animals and human subcutaneous white adipose tissue, with a relative lower levels in brown adipose tissue and much lower level in the brain.^10^ Moreover, it has been shown that Metrnl is down-regulated in white adipose tissues after a caloric restriction in animals (rats) while is dramatically up-regulated during white adipocyte separation.^15^ These data suggest that to understand the role of Metrnl in white adipose biology and metabolic homeostasis, there is a need for further research. Some studies demonstrated an insulin-sensitizing role of Metrnl in genetically engineered mouse.^11,15^ Tested by different methods, insulin resistance induced by a High Fat Diets (HFD) was impaired in adipocyte-specific Metrnl knockout subjects; otherwise transgenic mice with an overexpressing Metrnl in adipocytes were protected from diet-induced insulin resistance. The overexpression of Metrnl in adipose tissue is also different from insulin resistance in mice with leptin deficiency.^11^ The present study provided evidence that testifies adipose Metrnl is likely to improve overall insulin resistance on local adipose tissues in both autocrine and paracrine fashion.^11^ These researchers explained their evidence as follows: Firstly, although the phenotypes regarding insulin resistance in mouse models were obvious and clear, their serum Metrnl concentrations did not change compare to the control group. Secondly, the insulin-stimulated phosphorylation of AKT was boosted by adipocyte Metrnl in white adipose tissue, but not in all other major metabolic tissues such as brown adipose tissue, muscle and liver. Lastly, if circulating Metrnl levels increased with the intravenous administration of recombinant Metrnl for one week is unable to modify insulin resistance in adipose-specific Metrnl knockout mice fed with HFD. In addition to this, if acute intravenous injected with recombinant Metrnl could not improve hypoglycemic action in HFD-fed C57 obese mice or in leptin knockout obese mice.^11^

Altogether, these evidences suggest that the up-regulation of intramuscular Metrnl induced by a regular type of aerobic exercise would be an effective pathway to suppress obesity, type 2 diabetes and insulin resistance by improving the metabolism in almost whole-body tissues. Although Metrnl is considered to act positively in the cases of obesity caused by consuming HFD over time, to our knowledge, there are few studies related to changes following of intramuscular and peripheral Metrnl levels induction by regular aerobic exercise. In a recent study, it has been suggested that muscle Metrnl could effectively reduce fat accumulation by the increase of Metrnl in adipose tissue after exercise, which could be a beneficial factor for reducing the risk of chronic obesit.^11,16^ Considering the importance of the role of Metrnl in reducing insulin resistance and adipose tissue, as well as its relation to cardiovascular risk factors such as LDL, HDL, cholesterol and triglyceride, increasing the probability of Metrnl through regular exercise can promote heath in obese and diabetic population. The study of Metrnl and exercise (with its different aspects, e.g., type, intensity, duration) may also provide new insight and important understanding about the connections between the two and possibly prevention of type 2 diabetes and related diseases. We encourage exercise physiology and immunology researchers to examine the theoretical constructs present here.

## Ethical approval

Not Applicable.

## Competing interests

There is no conflict of interest.

## Funding

There is no funding resource.

## Authors’ contributions

Study concept and design: AS. Drafting of the manuscript: AS, AbK, ArK. Critical revision of the manuscript for important intellectual content: SMT, FM, MS, ABA, HZ. Study supervision: AS, SMT, HZ.
